# Malocclusion and TMJ disorders in teenagers from private and public schools in Mexico City

**DOI:** 10.4317/medoral.18075

**Published:** 2013-02-05

**Authors:** Leonor Sánchez-Pérez, María E. Irigoyen-Camacho, Nelly Molina-Frechero, Patricia Mendoza-Roaf, Carlo Medina-Solís, Enrique Acosta-Gío, Gerardo Maupomé

**Affiliations:** 1 DDS, MSD, PhD Professor, Health Attention Department.Universidad Autónoma Metropolitana Xochimilco; 2 DDS, PhD. Universitary Center of Health Sciences. Universidad de Guadalajara; 3 DDS, MSD Professor. Universidad Autónoma del Estado de Hidalgo; 4 DDS,PhD. Chief of Microbiology Laboratory, School of Dentistry, Universidad Nacional Autónoma de México; 5 DDS, PhD Professor. Department of Preventive and Community Dentistry at the Indiana University/Purdue University at Indianapolis School of Dentistry, and The Regenstrief Institute, Inc., Indianapolis, Ind., USA

## Abstract

Objective: To identify, among Mexican teenagers from public and private schools, the frequency, severity of malocclusion and orthodontic treatment needs, and their possible association with temporomandibular joint disorders. Material and Methods: Fifteen-year-old students were recruited from public and private schools. Clinical findings were registered as follows: oral hygiene status with the Oral Hygiene Index-Simplified, malocclusion using the Dental Aesthetic Index (DAI), and TMJ disorders following WHO criteria. Negative binomial and logistic regression models were constructed for data analysis. Results: A total of 249 fifteen-year old students were included in the study (118 female 47.4%). 68% had a DAI score ≤ 25 (minor or no occlusal anomalies), 18% scored 26-30 (mild anomalies), 7% scored 31-35 (evident anomalies), and 6% scored ≥ 36 (major malocclusion). The most frequent anomalies were dental crowding in 50%, maxillary dental irregularity in 44.6%, mandible irregularity in 41.2% and excessive maxillary overjet in 37.8%. Among the students, 26.1% had clicking/muscle or TMJ pain, of these 12.3% showed pain during palpation. OHI-S > 1 was found in 34% of the participants. The negative binomial model showed an association between DAI score and TMJ disorders (P=0.041). Also the logistic regression model showed an association between malocclusion (DAI>25) and TMJ disorders (OR=2.58, p=0.002). Malocclusion was associated also with poor oral hygiene (OR=1.65, p=0.007), and with attendance to public schools (OR=1.97, p=0.039). Conclusions: TMJ disorders and DAI scores were significantly associated. Screening/Diagnostic programs for ortho-dontic and TMJ-disorders are needed, to identify and offer treatment to teenagers with major malocclusion and TMJ/muscle pain.

** Key words:**Dental Aesthetic Index, DAI, crowding, Temporo Mandibular Joint disorders (TMJ disorders), Temporo Mandibular Disorders (TMD), occlusal anomalies, OHI-S.

## Introduction

The mouth’s appearance (including severe mal position of the teeth) may influence the quality of life in children and adolescents (1). The reported prevalence of malocclusion varies from 30% to 93% (2,3). In 1985, the World Health Organization (WHO) reported that 21% to 64% of children 13 to 14 years-old had orthodontic treatment needs (2).

The World Health Organization recommends the Dental Aesthetic Index (DAI) as a simple method to assess malocclusion severity and orthodontic treatment needs (4-6). The DAI consists of 10 occlusal traits related to dentofacial anomalies according to the three components: spacing, crowding and occlusion.

Malocclusion may be associated with temporomandibular joint (TMJ) disorders which cause orofacial pain and discomfort. Reported TMJ disorder prevalence varies from 2% in Turkish patients to 68% in young Polish adults (7-8). Associations have been reported between TMJ disorders and craniofacial anomalies such as open bite, cross-bite, molar distalization, or excessive overjet (9-11).

The use of dental care services by Mexican and Brazilian children and adolescents is affected by: health insurance, age, sex, parents’ schooling, race, socioeconomic variables, and oral health needs (12,13). In particular, orthodontic treatment is usually long and costly and may force parents to forgo prescribed orthodontic treatment for their children (13).

The objective of the present study was to identify the frequency and severity of malocclusion, orthodontic treatment needs and their association with the presence of temporomandibular joint (TMJ) disorders among Mexican teenagers from private and public schools.

## Material and Methods

This study was reviewed and approved by the committee for the protection of human subjects of research at the Universidad Autónoma Metropolitana.

- Study Group. A convenience sample of fifteen year old students was selected in two public (State supported), and two private schools in southern Mexico City. A n=240 sample size was estimated for alpha= 0.05, power= 0.80 and an estimated 30% prevalence of malocclusion minimal detectable odds ratio OR =2.3, (14).

The study initially targeted 300 participants; the schools’ authorities sent information on the study for reading and approval by parents or guardians. 270 (90.0%) signed consent forms were returned.

Additionally, each adolescent verbally assented to participate in the study. Twenty-one (7.8%) students 13 (11.4%) from private and 8 (5.13%) from public schools, p=0.057) were excluded because of current or past orthodontic treatment.

- Clinical Examination. Two examiners, experienced in oral epidemiology surveys and field-work, adhered to the World Health Organization (WHO) DAI standards and guidelines using the extended criteria and questionnaire on dento-facial anomalies. Their inter-examiner agreement for the 10 conditions included in DAI was kappa > 90%, and >92% for signs of TMJ disorder. DAI component scores were multiplied by a specific weight described by the WHO, and a constant was added to obtain a final DAI score for each student.

The oral examination was performed under artificial light with a flat dental mirror and a WHO dental probe. Recorded observations included diastemas, anterior open bite, as well as maxillary and mandibular anterior irregularities and overjet.

The WHO criteria were followed to evaluate symptoms and signs indicative of TMJ disorders. On the day of the clinical examination, participants were asked specific questions on the presence of TMJ symptoms, presence of clicking, pain or difficulty to opening and closing the jaw one or more times per week. Examiners recorded clinically assessed clicking, tenderness or reduced mobility of the jaw. Clicking was evaluated directly by an audible sharp sound or by palpation of the TMJ. Pain or muscular tenderness was evaluated by unilateral palpation with firm pressure of two fingers, exerted on the most voluminous part of the muscle. Tenderness was recorded only if the palpation spontaneously provokes an avoidance reflex. Reduced mobility is considered to be reduced if the student was unable to open the jaw to the width of two fingers.

Oral hygiene was evaluated using the Oral Hygiene Simplified Index (OHI-S). (15)

- Statistical Analysis. Bivariate statistics revealed no significant differences between the two private schools or between the two public schools. Therefore, the clinical findings were pooled for analysis into two groups: public or private schools. Categorical variables were compared with χ2 and Fisher´s exact test, as appropriate.

A negative binomial regression model was constructed using the DAI score as a dependent variable versus sex, school type and TMJ signs and symptoms as independent variables. The alpha coefficient was estimated to determine whether the data fitted a Poisson or a negative binomial distribution. Nominal logistic regression models were built using two DAI cut-off points >25 (classified by WHO as manifested malocclusion), and also using a higher cut-off point (DAI >28) as dependent variable, and sex, oral hygiene, and type of school as independent variables. Additionally, a nominal logistic regression model was constructed using TMJ signs as a dependent variable, and malocclusion and sex as independent variables. A value of p<0.05 was considered statis-tically significant. Hosmer and Lemeshow’s test was used to ascertain the goodness of fit of the model, (p>0.05). Data were analyzed using the statistical software JMP 8 (SAS Institute Inc., Cary, NC, USA).

## Results

A total of 270 students were examined, 21 were excluded in the present study because of current or past orthodontic treatment. 249 participants were included (131 boys, 118 girls), from public (n=148) and private schools (n=101). No significant differences across type of school were detected in the majority of the occlusal traits evaluated ( Table 1). Most (78.7%, n=196) students had at least one DAI anomaly. No difference was observed between boys and girls.

**Table 1:**
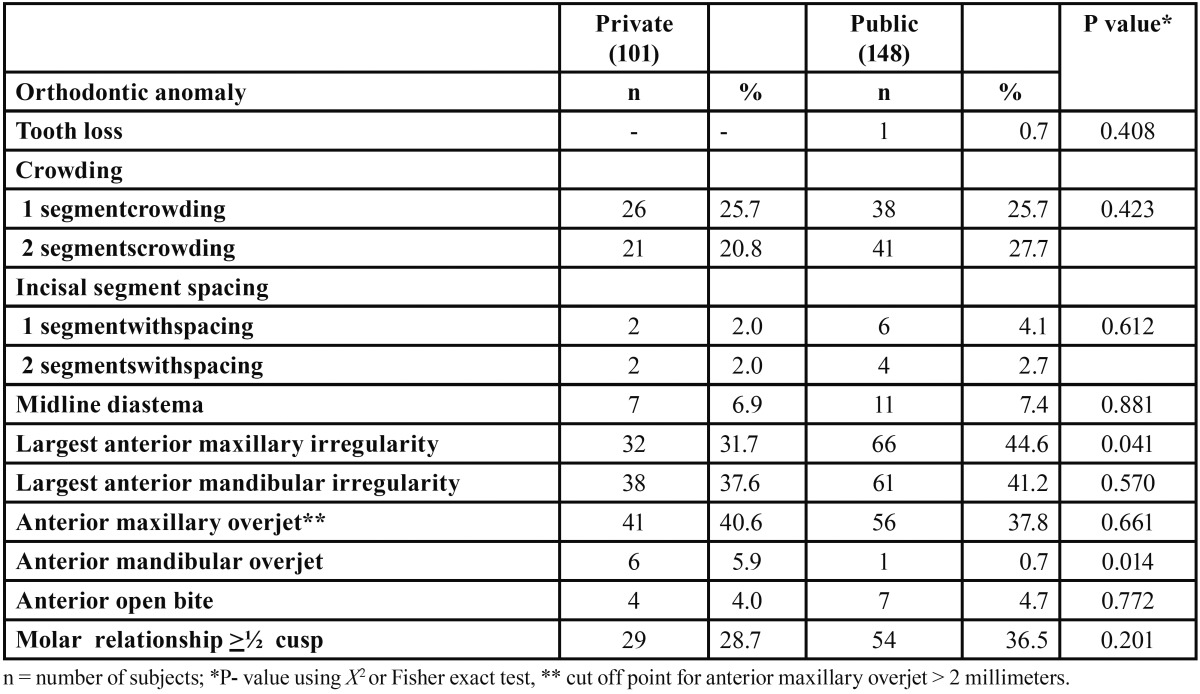
Prevalence of occlusal conditions by type of school.

Half of the teenagers showed dental crowding in one or two segments. Most had no space in the incisal segment, only 14 (5.6%) had one or two arch segments with interdental spaces. Only 18 (7.2%) adolescents presented diastemas, 39% had maxillary irregularities. Anterior maxillary and mandibular irregularities were significantly associated (p<0.001). Among these adolescents 61% had a maxillary overjet ≤ 2 mm, and 39.0% had an overjet >2 mm. Only 7 (2.8%) adolescents had a mandibular overjet between 1 to 4 mm. Eleven participants (4.5%) showed anterior open bite. Two thirds (n=166) had a normal molar relation, 61 (24.5%) had half cusp deviation, and 22 (8.8%) had full cusp deviation.

**Figure 1:**
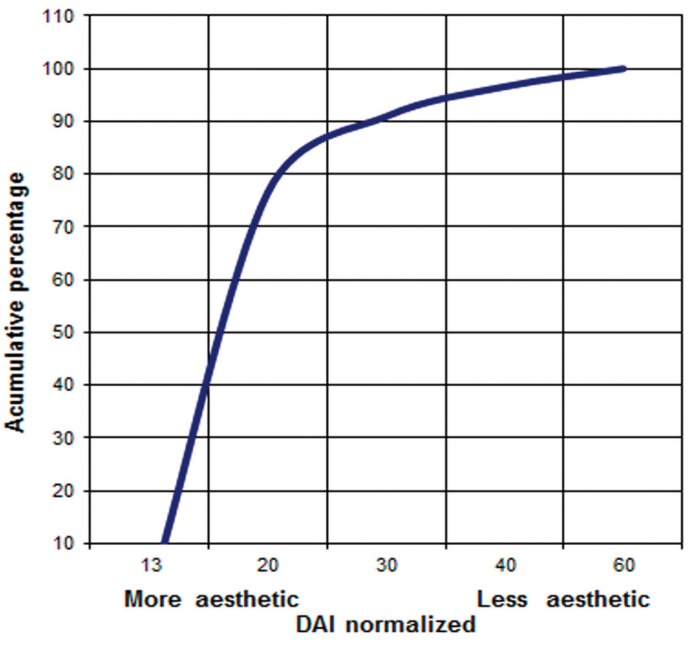
Distribution of normalized DAI in a group of Mexican teenagers.

Most (86%) students had low DAI scores (Fig. 1). DAI scores and orthodontic treatment needs are presented in  Table 2, among the students with malocclusion (n=80), 24% showed severe malocclusion and 19.0% very severe malocclusion. Average DAI was 22.1 (standard deviation ± 7.2). No statistically significant differences were observed between boys and girls (p=0.537) or across private and public (p=0.241) schools 

**Table 2:**
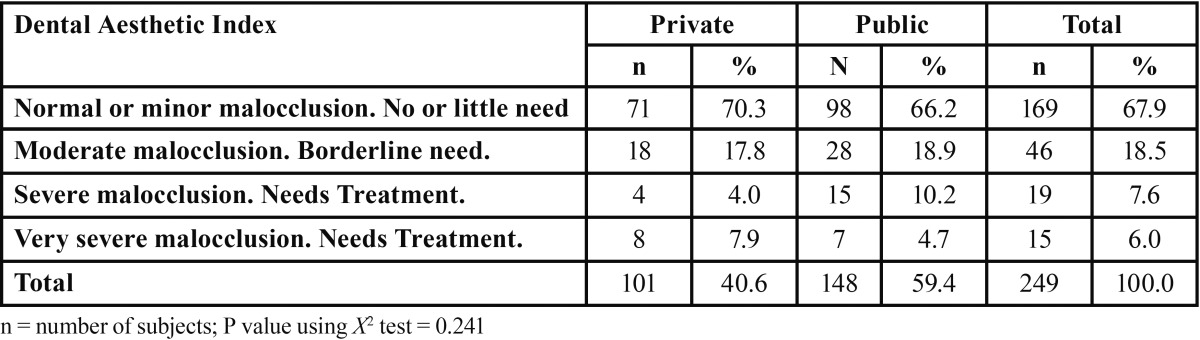
Distribution of DAI across type of school.

No reduced mandibular opening ability was observed but 26.1% of subjects showed TMJ clicking or pain during palpation, among them 12.3% had pain and the rest clicking. Of all the students, 3.2% experienced pain during palpation, in this group 87.5% were females and 12.5% males (p<0.029).Pain and malocclusion were associated as 75% of the students with pain had a high DAI score (p<0.015). TMJ symptoms were referred by few (6.5%) students, who reported TMJ clicking or pain one or more times a week, most of them (98%) presented also TMJ clicking or pain during oral examination (p<0.0001). A significant (p=0.015) relationship was found between mandibular overjet and TMJ signs also tooth crowding in both arches was more frequent among students with TMJ signs (p=0.046). Other occlusal traits, considered in the DAI, were not significantly associated with TMJ alterations.

Mean OHI-S was 0.6 (± 0.8), with 34% of students scoring higher than 1. Girls had a significantly (p<0.001) lower mean OHI-S (0.40) than boys (0.80).

TMJ alterations were significant in the negative binomial regression model for malocclusion adjusted by sex, type of school, and oral hygiene ( Table 3). The expected DAI score was increased by 1.86% in teenagers with TMJ alterations. The alpha coefficient 0.038 indicated that the binomial distribution (p<0.001) was better than the Poisson distribution to model the data.

**Table 3:**
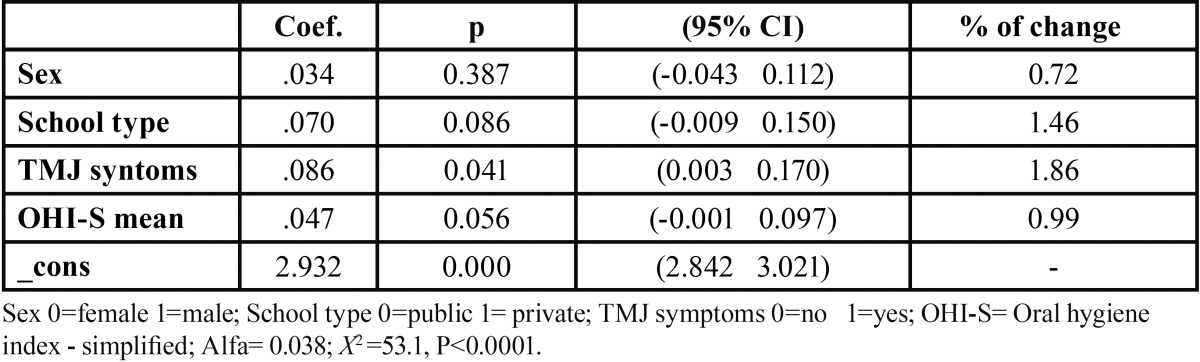
Results of the negative binomial regression model fitted for DAI score and sex, school type, TMJ symptoms and oral hygiene.

The nominal logistic regression model, fitting malocclusion (DAI ≥ 25) and TMJ status revealed an association between these two conditions (OR=2.68, p=0.037), also DAI ≥ 25 was associated with poor/fair oral hygiene (OHI-S>1) and with attendance to public schools (OR= 1.97, p= 0.039) In the logistic regression model for malocclusion using a higher cut-off point (DAI ≥ 28) similar associations were detected by type of school (OR=1.95, p=0.036), level of oral hygiene (1.79, p=0.004), and TMJ disorders (2.18, p=0.019), ( Table 4),

**Table 4:**
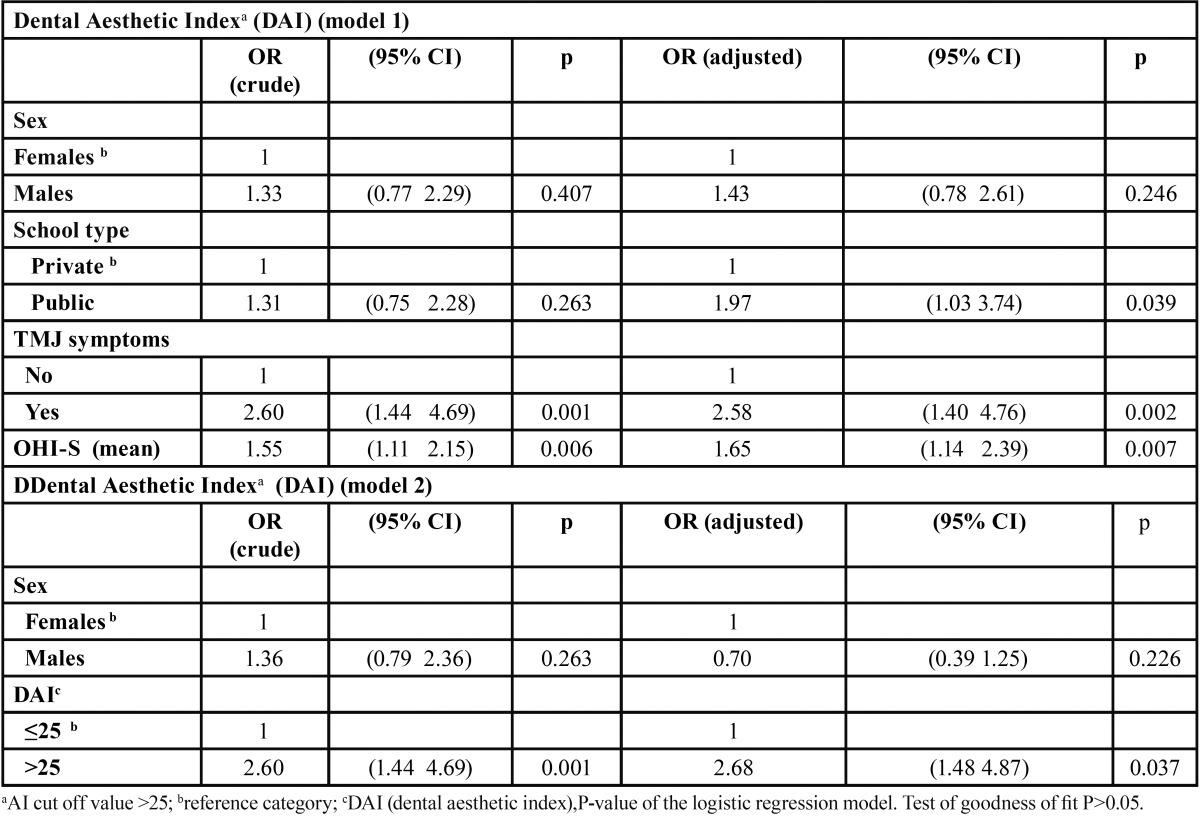
Results of the logistic regression models of Dental Aesthetic Index (model 1) and for temporomandibular joint disorders (model 2) and selected variables.

## Discussion

The present report is, to our knowledge, the first survey in Mexican adolescents using the Dental Aesthetic Index (DAI) and is the first description of frequency and distribution of malocclusions and TMJ disorder in Mexican adolescents. Almost one third of the students presented occlusal anomalies leading to orthodontic treatment needs; among them 24% had severe occlusal anomalies, and 19% very severe anomalies. Diagnostic and treatment programs may be necessary to treat the students particularly those with severe malocclusions.

Crowding of teeth was the anomaly with the highest prevalence in this study (50.6%). Normal occlusion is characterized by the continuity of the arch (2,16).Some factors contribute to the maintenance of this continuity (interproximal contact, transseptal fibers, and the direction of occlusal forces) (17), while others may be detrimental, such as early tooth loss and microdontia (18).Our data support the WHO observation that dental crowding is the most prevalent dental anomalies and is closely associated with dental plaque retention (19).

The largest anterior maxillary and mandibular irregularity may be rotations or displacement with respect to the normal alignment. In the present study, 40% of the subjects presented this anomaly, one of the four more frequent conditions. This prevalence was higher than some reports (18,20) but similar by others (19,21-23). The difference in the prevalence of this trait could be attributed to crowding predisposition caused by development and maturation of the arches, deleterious oral habits, and discrepancies of jaws or environmental factors which varied among the different groups studied.

Sixty-one percent of the adolescents presented between 0 and 2 mm of maxillary superimposition in a horizontal relation, and 40% presented a 3-8 mm overjet. With a cut-off value of 4 mm, only 13% of the students had excessive overjet. Mandibular superimposition was detected in 2.8% of the teenagers. Only 4.4% of the adolescents in this study presented anterior open bite. The results of this study are similar to some reports (21,23), but higher than others (18-20). Only Thilander (17), reported almost 7% prevalence among Colombian students.

The 24.4% of adolescents presented a half cusp deviation between the first two upper and lower molars in centric relation, and 8.8% presented a full cusp deviation. Similar profiles have been reported for most populations, with some exception (20,22-23). Differences in prevalence may be due to variation in growth and development, oral habits, tongue interposition, bone discrepancies, genetic predisposition, and/or environmental factors (2,17,18).

The mean DAI score was 22.1, no significant differences were observed between boys and girls in their orthodontic treatment needs. Some authors suggested the DAI scores are useful to identify patients who must be referred to specialist care, thus accruing an important advantage to public health programs (4,23).

Of the students, 6.5% reported TMJ clicking or pain one or more times a week and 26.1% presented TMJ alterations during the oral examination, in this group 12.3% showed pain during palpation of the TMJ area, pain was more frequently found among females. Women appear to show a higher risk of TMJ muscular tenderness or pain than males; hormonal, behavioral or psychological differences have been considered as possible etiological factors of this finding (24).

There was an association between TMJ signs and symptoms reported and the findings during oral examination, more than 90% of the children who reported TMJ symptoms showed signs of TMJ problems during the oral examination. A longitudinal study on adolescent women in Japan found that a history of sounds was related to the subsequent development of symptoms (25), suggesting the importance of early diagnosis.

An association between malocclusions (DAI score) and TMJ sing and symptoms was detected, as Thilander et al., (9) found among Colombian children and adolescents (excessive overjet and Angle Class III). In the present study the individual characteristics included in the DAI showing significant association with TMJ disorder were anterior crowding and maxillary overjet. A longitudinal study in US children followed from 7 to 15 years of age found that mesial molar occlusion at age 15 was significantly related to TMJ disorders (26). In this study large overjet was the only morphological variable which seemed to consistently increase the risk of TMJ disorders.

The results of the Mexican adolescents studied showed an association between TMJ disorder and the high score of DAI, which involves the presence of different occlusion anomalies. Current criteria consider that while orthodontic treatment cannot be used to prevent TMJ disorder, it may be advantageous to use it in certain patients that have already developed TMJ signs and symptoms (27).

The results of the multiple logistic regression model indicated that students in public schools (who usually have a lower socioeconomic status –SES- than those in private schools) had higher frequency of malocclusions in association with poorer oral hygiene. Socioeconomic disparities in oral health have been found in other studies (28). In our study population we may speculate that students from private schools receive more dental care than children from public schools, thereby leading to earlier or more appropriate interventions which might reduce malocclusion risk. Of twenty-one students with current or past orthodontic treatment, 13 (11.4%) were from private and 8 (5.13%) from public schools (p=0.057).

Some reports suggest that the type of school is a surrogate indicator for socioeconomic status in the Latin-American context (29). In Mexico, children from public schools had a higher risk of seeking dental care associated with odontalgia than children attending private schools (30).

Methodological limitations. Direct comparisons of our findings with those of recent epidemiological reports on malocclusions and treatment needs in various countries, is hindered by diversity of criteria, and cut-off points.

The group of students selected did not represent the 15-year-old adolescents in Mexico City, because less than 60% of the 15-year olds attend junior high school. Public education is offered at no-cost in Mexico, whereas private education may cost a substantial tuition fee. In the area studied only 18.5% of the teenage students attend private schools.

The cross-sectional design of this study is a limitation to ascertain causes and effects. In addition, DAI does not consider alterations such as posterior cross bite, or midline deviations, among others, which might have an impact on the analysis of the association of malocclusion with different study variables. Incorrect malocclusion classification may have underestimated the strength of associations. However, this first description of frequency and distribution of malocclusions and TMJ disorder on Mexican adolescents contributes useful information that point to the general direction for further research and interventions.

## Conclusions

The current study showed that almost one third of the students presented occlusal anomalies leading to orthodontic treatment needs and more than a quarter of the participants showed TMJ disorder. An important percentage of the students presented dental anomalies that affect their appearance to various degrees of severity; such disadvantages might compromise the well-being of these young people, in particular those with lower SES who have been shown to be differentially affected compared to children with higher SES. Screening/Diagnostic programs for orthodontic and TMJ-disorder are needed, to identify and offer treatment to teenagers with major malocclusion and TMJ/muscle pain.
